# Investigation of copper(II) tetrafluoroborate catalysed epoxide opening

**DOI:** 10.1016/j.tetlet.2011.10.090

**Published:** 2011-12-28

**Authors:** Amy S. Capes, Arthur T. Crossman, Lauren A. Webster, Michael A.J. Ferguson, Ian H. Gilbert

**Affiliations:** Division of Biological Chemistry and Drug Discovery, College of Life Sciences, University of Dundee, Sir James Black Centre, Dundee DD1 5EH, UK

**Keywords:** Epoxide, Copper(II) tetrafluoroborate, Lewis acid, Alcohols

## Abstract

We report the extension of the copper(II) tetrafluoroborate catalysed opening of epoxides with alcohols to include a wider variety of alcohols, a range of solvents and a method to purify the products from the reaction.

## Graphical abstract

**Figure:**
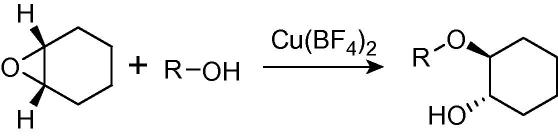

Ring-opening reactions of epoxides provide a synthetically important path to a variety of β-substituted alcohols.^1,2^ β-Alkoxyalcohols and β-aminoalcohols in particular are important moieties in many biologically active molecules. During the course of our work, it was necessary to carry out ring-opening of cyclohexene oxide with an alcohol to give the β-alkoxyalcohol derivative.

The ring-opening of cyclohexene oxide with an alcohol almost always employs a Lewis acid catalyst as a promoter in order to avoid the use of strong acids or bases and heating. A number of different Lewis acid catalysts have been reported.^3–22^ Although some of these Lewis acids give good yields under mild conditions, they often suffer from problems such as lack of commercial availability, toxicity, use of expensive metals, or the need for large amounts of catalyst.

Recently, hydrated copper(II) tetrafluoroborate has been reported as an efficient catalyst for ring-opening reactions of epoxides, including cyclohexene oxide, with alcohols under mild conditions (Scheme 1).^23^ This catalyst has the advantage of being cheap and commercially available. In addition, the ring-opening reaction yields only *trans* diastereomers. The reaction has also been reported under microwave conditions,^24^ and using amine nucleophiles, although in this case a solvent-free system was used.^25^ However, the reaction has some drawbacks. The optimum yields are obtained with four equivalents of alcohol, which makes purification difficult when nonvolatile alcohols are used. The amount of alcohol required is also problematic when it is the most valuable reagent. The scope of the reaction is further restricted by the solubility of reagents, because the only reported solvent for the reaction is dichloromethane.

The reaction has previously been reported using common alcohols, such as methanol, isopropanol, *tert*-butanol, allyl alcohol and benzyl alcohol, which do not provide much opportunity for further synthesis. It was decided to expand the scope of the reaction, both in terms of what substrates are suitable, and synthetic utility. We present further synthetically useful substrates for this reaction, a new method for separating the products from excess alcohol and a study of alternative solvents.

Initially, work focused around opening the epoxides with mono-protected diols and N-protected alkyl amino alcohols (entries 1–4); these gave the desired products in reasonable yields (Table 1). These protected alcohols can be further functionalised in a wider synthesis. The O-protected amino alcohol (entry 5) did not react, possibly because the primary amine coordinated to the copper catalyst; the copper(II) tetrafluoroborate catalysed amine epoxide opening has only been reported in solvent-free versions of this reaction.^25^ A number of other functionalised alcohols were investigated (Table 1). For some of them the reaction worked well, however in other cases the yields were poor. The poor solubility of some of the substrates in CH_2_Cl_2_ is likely to have contributed to the failure of the reaction in at least some cases (entries 7, 8, 12 and 13).

The only solvent recorded in the literature for this procedure is CH_2_Cl_2_, therefore the effect of solvent on the reaction was investigated. The ring-opening reaction with TBDPS-protected butane-1,4-diol was used because this was one of the few examples where the product could be purified directly (Scheme 2). In addition, the alcohol is also readily soluble in all the solvents used in the study.

The CH_2_Cl_2_ control reaction worked with TBDPS-protected butane-1,4-diol (Table 2, entry 2) to give a yield comparable to previous reactions (∼60%), indicating that the results under these conditions can be usefully compared to previous findings. The three chlorinated solvents tested showed similar yields, with chloroform and CH_2_Cl_2_ giving slightly higher yields than DCE. Solvents containing a carbonyl group (EtOAc, DMF, NMP) generally gave poor yields, with only EtOAc giving a minimal amount of product. Similarly MeCN only gave a low yield. The solvents containing an ether functionality gave yields that were generally lower than the chlorinated solvents, although diethyl ether performed almost as well as CH_2_Cl_2_. However, CMPE and THF were low yielding and 1,4-dioxane gave no reaction at all. Toluene gave the highest yield of all the solvents tested.

There are several possible reasons for nonpolar solvents increasing the rate of reaction. Firstly, polar solvents with readily available lone pairs, such as DMF, NMP and 1,4-dioxane, could potentially compete with the epoxide in co-ordinating to the copper catalyst, leading to a reduced rate of reaction. In contrast, solvents with no lone pairs, such as the chlorinated solvents and toluene do not complex strongly to the copper and do not prevent the epoxide binding. Secondly, if the rate determining step is the attack of alcohol on the copper catalyst epoxide complex via an S_N_2 mechanism, then the transition state complex will have a more distributed charge relative to the reactants and hence is more stabilised by a nonpolar solvent (Scheme 3). In addition, both reactants would have a higher degree of solvation in a polar solvent and therefore the nucleophile would be less available to attack the epoxide.^27–29^

Although the solvent must allow some of the copper catalyst to dissolve, it was observed that the negative effects of a polar solvent far outweighed the benefit of having more catalyst in solution. However, some of the more polar but lower yielding solvents may be useful where substrate solubility is an issue. These results show that there are several other solvents that are suitable for this reaction, which may extend the type of substrates that can be used. There is certainly more scope for exploring solvents for this reaction.

A further complication arises because the reaction works best with four equivalents of alcohol. In a number of instances, the product and the alcohol reagent had similar polarity, which made separation of the product and the alcohol problematic. Previously, this problem has been overcome either by using volatile alcohols which could be removed in vacuo, by washing with water,^24^ or by acetylating the reaction mixture before performing column chromatography, and then deacetylating the purified material.^30^

The first two options were not available because none of the alcohols used in this study are volatile or water soluble, and the third option is laborious because it adds two steps to a synthesis. We developed an alternative and shorter procedure using trityl chloride to remove the excess of primary alcohol substrate, leaving the secondary alcohol product untouched. The crude reaction mixture was treated with trityl chloride, allowing facile chromatographic separation of the product from the reagents and removing the need for a further deprotection step (Scheme 4), thereby shortening the synthetic procedure. The required product was obtained in 78% yield over two steps with this method, which was used for all compounds in Table 1, except for Table 1, entry 4.

In conclusion, a novel selection of alcohols has been used to extend the scope of the Cu(BF_4_)_2_ catalysed epoxide opening reaction. A solvent study has shown several alternatives to CH_2_Cl_2_, which may allow new substrates to be used with the reaction. Furthermore, a method for removal of excess primary alcohol in a single, simple step has been outlined.

## Solvent study ring opening method for *trans*-2-{4-[(*tert*-butyldiphenylsilyl)oxy]butoxy}cyclohexanol

Cyclohexene oxide (50 μL, 0.495 mmol, 1 equiv), 4-[(*tert*-butyldiphenylsilyl)oxy]butan-1-ol^31^ (651 mg, 1.982 mmol, 4 equiv) and Cu(BF_4_)_2_ (14 mg, 0.071 mmol, 0.1 equiv) were dissolved in the solvent (3 mL). The reaction mixture was stirred under argon overnight at room temperature, diluted with CH_2_Cl_2_ (5 mL) then washed with H_2_O (15 mL), and passed down a phase separator. The solvent was removed in vacuo, and the crude purified by radial-band chromatography (100% hexane to 10:1 Et_2_O/hexane). The identity of the product was confirmed by NMR.

^1^H NMR (500 MHz, CDCl_3_) *δ*_H_ 7.68–7.65 (4H, m), 7.44–7.36 (6H, m), 3.71–3.60 (3H, m), 3.42–3.32 (2H, m), 3.07–2.96 (1H, m), 2.42 (1H, br s), 2.06–1.98 (2H, m), 1.72–1.60 (6H, m,), 1.28–1.17 (4H, m), 1.05 (9H, s) ppm. ^13^C NMR (125 MHz, CDCl_3_) *δ*_C_ 135.6 (CH), 134.0 (C), 133.7 (C), 129.7 (CH), 129.6 (CH), 127.7 (CH), 127.6 (CH), 83.7 (CH), 73.8 (CH), 68.6 (CH_2_), 64.0 (CH_2_), 63.7 (CH_2_), 62.8 (CH_2_), 32.0 (CH_2_), 29.9 (CH_2_), 29.3 (CH_2_), 29.2 (CH_2_), 26.9 (CH_3_), 26.7 (CH_3_), 24.3 (CH_2_), 24.0 (CH_2_), 19.3 (C) ppm. HRMS calcd mass for C_26_H_39_O_3_Si (M+H^+^): 427.2663, found: 427.2645 (4.3 ppm).

## General ring-opening method, as exemplified by *trans*-2-{3-[(*tert*-butyldiphenylsilyl)oxy]propoxy}cyclohexanol (Table 1, entry 3)

Cyclohexene oxide (0.159 mL, 1.574 mmol, 1 equiv), 3-[(*tert*-butyldiphenylsilyl)oxy]propan-1-ol (1.980 mg, 6.296 mmol, 4 equiv) and Cu(BF_4_)_2_ (4 mg, 0.016 mmol, 0.01 equiv) were dissolved in CH_2_Cl_2_ (10 mL) using sonication (1 min). The mixture was stirred under argon overnight at room temperature. The mixture was diluted with H_2_O (20 mL), extracted with CH_2_Cl_2_ (3 × 15 mL) and then the combined organic extracts were washed with brine (50 mL) and filtered through cotton wool. The solvent was removed to leave a residue.

## General tritylation method as exemplified by *trans*-2-{3-[(*tert*-butyldiphenylsilyl)oxy]propoxy})cyclohexanol

The crude mixture was dissolved in pyridine (4 mL) and added to TrCl (1.755 g, 6.296 mmol, 4 equiv). The resulting mixture was stirred at 70 °C for 16 h. The pyridine was removed by co-evaporation with toluene and the residue resuspended in EtOAc (50 mL). The organic phase was washed with H_2_O (50 mL), and the aqueous layer extracted with EtOAc (2 × 50 mL). The combined organic extracts were washed with H_2_O (100 mL) and brine (100 mL) and then filtered through cotton wool. The solvent was removed and the crude purified by column chromatography (Et_2_O/hexane, 0:100 to 2:3) to afford the product as a clear oil (531 mg, 78%).

^1^H NMR (500 MHz, CDCl_3_) *δ*_H_ 7.67 (4H, dd, *J* = 7.8, 1.4 Hz), 7.43–7.35 (6H, m), 3.80–3.74 (3H, m), 3.53–3.48 (1H, m), 3.41–3.36 (1H, m), 3.03–2.98 (1H, m), 2.07–1.98 (2H, m), 1.81 (2H, quin, *J* = 6.2 Hz), 1.72–1.67 (2H, m), 1.29–1.09 (4H, m), 1.05 (9H, s) ppm. ^13^C NMR (125 MHz, CDCl_3_) *δ*_C_ 135.6 (CH), 133.9 (C), 133.9 (C), 129.6 (CH), 127.7 (CH), 127.6 (CH), 83.7 (CH), 73.8 (CH), 65.3 (CH_2_), 60.8 (CH_2_), 33.1 (CH_2_), 32.0 (CH_2_), 29.2 (CH_2_), 26.9 (CH_3_), 24.3 (CH_2_), 24.0 (CH_2_), 19.2 (C) ppm. HRMS calcd mass for C_25_H_37_O_3_Si (M+H^+^): 413.2506, found: 413.2498 (2.1 ppm).

## Figures and Tables

**Scheme 1:**
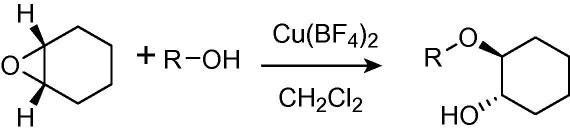
General reaction for copper(II) tetrafluoroborate catalysed epoxide opening.

**Scheme 2:**
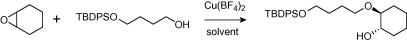
Copper(II) tetrafluoroborate catalysed epoxide opening solvent study.

**Scheme 3:**
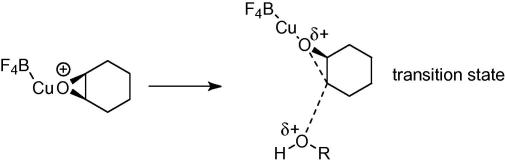
Transition state for the copper(II) tetrafluoroborate catalysed opening of cyclohexene oxide.

**Scheme 4:**
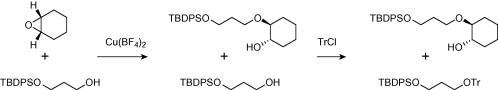
Example of the trityl chloride method for removing excess alcohol.

**Table 1:** Reaction time and yield^a^

Entry	Alcohol/amine	Yield (%)	Entry	Alcohol	Yield (%)
1		66	8		0
2		53	9		60
3		78	10		b
4		63	11		65
5		0	12		0
6		53	13		0
7		0			

a Reactions carried out using 1% catalyst and 4 equiv alcohol at room temperature for 24 h in CH_2_Cl_2_.

b Unidentified product.

**Table 2:** Effect of the solvent on the reaction^a^

Entry	Solvent	Yield (%)	Snyder polarity index^b^
1	CHCl_3_	62	4.1
2	CH_2_Cl_2_	58	3.1
3	DCE	46	3.5
			
4	EtOAc	16	4.4
5	DMF	0	6.4
6	NMP	0	6.7
			
7	MeCN	10	5.8
			
8	Et_2_O	54	2.8
9	CPME^c^	38	Not available
10	THF	21	4.0
11	1,4-Dioxane	0	4.8
			
12	Toluene	66	2.4

a Monitored by TLC, product isolated by radial-band chromatography and the structure confirmed by NMR spectroscopy.

b Polarity is indicated by the Snyder Polarity Index.^26^

c CMPE-cyclopentyl methyl ether.
